# *Helicobacter* *pylori* Infection and Antimicrobial Resistance Surveillance over 25 Years in Children in Gipuzkoa, Northern Spain

**DOI:** 10.3390/microorganisms14020389

**Published:** 2026-02-06

**Authors:** Itsaso Jimenez, Iñigo Ansa, Pedro Vallejo, Milagrosa Montes

**Affiliations:** 1 Microbiology Department, Donostia University Hospital, Osakidetza Basque Health Service, Paseo Dr Beguiristain s/n, 20014 San Sebastián, Gipuzkoa, Spain; itsasojim@yahoo.es (I.J.); inigo.ansaisasa@osakidetza.eus (I.A.); 2 Microbiology Department, Biogipuzkoa Health Research Institute, Donostia University Hospital, Osakidetza Basque Health Service, 20014 San Sebastián, Gipuzkoa, Spain; pedro.vallejorecuna@osakidetza.eus

**Keywords:** *Helicobacter pylori*, children, biopsy, resistance, clarithromycin, metronidazole%

## Abstract

The diagnosis and treatment of *Helicobacter pylori* infection in children represent a major public health problem worldwide, and although prevalence in developed countries has declined, concern about antibiotic resistance continues to grow. This retrospective study aimed to analyze *H. pylori* infections in children under 15 years of age in Gipuzkoa (Basque Country, Spain) over a 25-year period and to describe patterns of primary and secondary antimicrobial resistance. All diagnostic tests received at a tertiary referral hospital between 2000 and 2024 were reviewed: urea breath test, gastric biopsy culture, string test, and stool antigen detection. Antimicrobial susceptibility testing was performed using the E-test gradient diffusion method. Out of 2855 children investigated, 26.9 were infected with *H. pylori*. Eradication control was performed in 62.5% (*n* = 480) of cases. Antimicrobial susceptibility testing was achieved in 96.5% out of the 400 isolates studied. All isolates were sensitive to amoxicillin and tetracyclines. Primary resistance to clarithromycin, metronidazole, and levofloxacin was 28.9%, 19.4%, and 8.9%, respectively, and secondary resistance was 38.7%, 29.7%, and 5.9%, respectively. *H.pylori* infection remains a challenge in pediatric patients, and the high resistance observed to clarithromycin and metronidazole makes it necessary to monitor the susceptibility of *H.pylori* and confirms the need for targeted treatment.

## 1. Introduction

*Helicobacter pylori* (*H. pylori*) infection is a major public health problem worldwide, and its prevalence varies greatly between different regions of the world, depending on the social, hygienic, and economic conditions of the countries [1]. According to data provided by Hooi et al., the regions with the highest prevalence, exceeding 60–70%, are Africa, South America, and Western Asia. In contrast, Oceania, Western Europe, and North America are among the regions with the lowest prevalence (24–38%) [2]. In developed countries, prevalence has declined in recent decades. In Japan, data show a drop from 10% in children born in 1985 to less than 3% in those born in 2011, evidencing a marked birth cohort effect due to improvements in hygiene and social conditions [3]. Nonetheless, *H. pylori* infection still represents a high disease burden, and thus antimicrobial resistance vigilance should not be ignored.

*H. pylori* infection in pediatrics has different clinical manifestations than in adults. The infection is mainly acquired in the first years of life and is, in most cases, an asymptomatic infection, with only a minority of those infected developing symptoms. At all ages, gastritis, followed by gastric and duodenal ulcers, are the most frequent manifestations. To a lesser extent, *H. pylori* causes gastric cancer and MALT (Mucosa Associated Lymphoid Tissue) lymphoma. In children, it is recommended to investigate *H. pylori* when peptic ulcer or complications (hematemesis, melena, unexplained iron deficiency anemia) are suspected, but not in any case of chronic abdominal pain without warning signs.

An update to the ESPGHAN (European Society for Pediatric Gastroenterology, Hepatology and Nutrition) and NASPGHAN (North American Society for Pediatric Gastroenterology, Hepatology and Nutrition) guidelines [4] for the diagnosis and management of *H. pylori* infection in children and adolescents was recently published (2024). The main updates to the previous 2017 guidelines [5] include not testing for *H. pylori* in children with functional abdominal pain consistent with brain–stomach disorders or in cases of growth retardation, chronic idiopathic thrombocytopenic purpura, inflammatory bowel disease, celiac disease, or eosinophilic esophagitis.

In children, unlike in adults, the initial diagnosis of *H. pylori* infection should be invasive: upper gastrointestinal endoscopy with biopsies for histology, a rapid urease test, and, if possible, culture or molecular techniques. The use of serology is not recommended, and non-invasive tests (fecal antigen, UBT) are reserved for confirming eradication after treatment [4].

The test and treat strategy in children is not recommended. Treatment indications in children are limited to patients with peptic ulcer, refractory iron deficiency anemia, or a first-degree family history of gastric cancer [6]. Eradication in children is complex because antibiotic options are more limited and because of the high rate of resistance. There are numerous publications warning of high resistance to antimicrobials used in the eradication treatments of *H. pylori,* in Spain, Europe, and worldwide [7,8,9]. Nevertheless, different regions have different resistance profiles that should be individually studied. Treatment should be based on targeted therapies (guided by culture and antibiogram) whenever possible and, if not, regimens should be used according to local resistance profiles, and eradication should always be confirmed. At our hospital, following the recommendations of consensus guidelines, all gastroduodenal biopsies from patients in whom *H. pylori* infection must be ruled out are cultured and all *H. pylori* isolates undergo antimicrobial susceptibility testing in order to provide targeted treatment whenever possible.

The objectives of the study were to report episodes of *H. pylori* infection in children under 15 years of age over a 25-year period and to determine primary and secondary resistance to the five most used antimicrobials for its eradication.

## 2. Materials and Methods

### 2.1. Ethics Statement

This study was undertaken with approval from the Ethics Committee of Donostia University Hospital (MGA-HEL-2022–01).

### 2.2. Patients and Samples

This is a retrospective study of *H. pylori* infection carried out in the Microbiology Department of Donostia University Hospital (DUH), Gipuzkoa, Basque Country, Spain, between 2000 and 2024. All samples received from patients < 15 years of age, whose pediatrician or gastroenterologist had requested testing to rule out *H. pylori* infection, were included.

DUH is the referral hospital for pediatrics for the entire province and treats virtually all children living in Gipuzkoa. The population attended to at DUH the first year of each period can be seen in Table 1 (https://es.eustat.eus).

*H. pylori* was investigated in different types of samples: gastroduodenal biopsies and string tests (Entero-test HDC Corp, Mountain View, CA, USA) for *H. pylori* culture, exhaled air for the urea breath test (UBT), and stool for antigen detection.

Each patient with documented *H. pylori* infection was considered as one incident case; detections of *H. pylori* after eradication failure in the same individual were not counted as a new case.

A followed-up case was defined as a patient who, at least one month after a positive diagnostic test, had a confirmatory (control) test to assess bacterial eradication. In patients with more than one therapeutic failure (i.e., more than one positive control test after successive eradication treatments), only the last recorded control test was considered. Those patients for whom no confirmatory test was recorded were classified as uncontrolled cases.

### 2.3. H. pylori Culture (Biopsy and String Test)

Biopsies were received in the laboratory in sterile containers with saline solution on the same day as the endoscopy.

The string tests were performed in the microbiology laboratory. The string was collected in an empty Petri dish and processed immediately afterwards [10,11]. This test was performed until 2013, when it was discontinued.

The biopsies and string tests were cultured following the conditions described by Gómez-Ruiz de Arbulo et al. [12]. Briefly, biopsies and strings were seeded in selective media, until 2013 on Brucella agar plates (BBL, Beckton-Dickinson, Madrid, Spain) supplemented with 5% hemolyzed horse blood and 1% Vitox (Oxoid Ltd., Basingstoke, UK), trimethoprim 5 micrograms/mL, and vancomycin, and subsequently on commercial selective plates for *H. pylori* (Pylori agar, bioMérieux). The plates were incubated under microaerophilic conditions (2% H_2_, 5% O_2_, 7% CO_2_, 86% N_2_) at 37 °C and 80% humidity for a minimum of 7 days before classifying a culture as negative.

All colonies suspected of being *H. pylori* have been identified using matrix-assisted laser desorption ionization–time of flight mass spectrometry (MALDI-TOF-MS, MALDI Biotyper^®^, Bruker Daltonics, Billerica, MA, USA) since 2015; previously, we used colony characteristics, Gram staining, and catalase, urease, and oxidase tests.

### 2.4. Antimicrobial Susceptibility

All cultured isolates have undergone an antibiogram using the E-test against amoxicillin (AMX), clarithromycin (CLA), metronidazole (MTZ), levofloxacin (LEV), tetracycline (TET), and rifampicin (RIF, since 2022).

Until 2012, the susceptibility criteria recommended by the National Committee for Clinical Laboratory Standards [13] were followed, and since then, the criteria published by the European Committee on Antibiotic Susceptibility Testing (EUCAST Clinical Breakpoint Tables v.9.0) (http://www.eucast.org/clinical_breakpoints/ accessed on 5 February 2026) have been followed. Isolates with CLA minimum inhibitory concentrations (MICs) > 0.5 mg/L, LEV MIC > 1 mg/L, MTZ MIC > 8 mg/L, AMX MIC > 0.125 mg/L, TET MIC > 1 mg/L, and RIF MIC > 1 mg/L were considered resistant.

In those isolates where the antibiogram could not be performed due to technical problems (contamination or because the isolate was not viable), susceptibility to CLA was determined by PCR amplification of the 23S gene, followed by sequencing to detect the most frequent mutations associated with macrolide resistance [14].

Primary resistance was determined considering the first gastroduodenal biopsy or string test from patients who had not undergone any previous diagnostic tests for *H. pylori* diagnosis in our laboratory (naïve patients). Secondary resistance was determined including data from biopsies and string tests performed on patients who had a previous *H. pylori*-positive test result, assuming they had undertaken an eradication therapy prior to the collection of the second or subsequent samples.

### 2.5. Urea Breath Test (UBT)

The UBT was performed according to the manufacturer’s instructions (TAU-KIT^®^ 100 mg soluble tablets 13C-Urea, ISOMED PHARMA, S.L. Las Rozas, Madrid, Spain). Briefly, the child takes 100 mL of Citral pylori^®^ solution dissolved in water. Ten minutes later, exhaled air samples are collected to determine the baseline value. The patient then drinks half a tablet (50 mg of 13C urea) dissolved in 50 mL of water. Thirty minutes later, exhalation samples are collected. The samples are measured by Isotope Ratio Mass Spectrometry; when the difference δ 13C Post-δ 13C Basal ≥ 5‰, the result is positive.

### 2.6. H. pylori Antigen in Stool

The test (Amplified IDEIA TM HpStAR. Oxoid Ltd., Hants, UK) is a sandwich enzyme immunoassay that uses highly specific monoclonal antibodies for detection of *H. pylori* antigen in feces. If the stool sample is liquid, 100 µL is used; if it is solid, approximately 0.1 g is homogenized with a buffer and 100 µL of the supernatant is used. The EIA was performed following the manufacturer’s instructions.

### 2.7. Statistical Analysis

Categorical data were compared using the χ^2^ test with InStat version 3.1 software. Differences were statistically significant when the *p*-value was less than 0.05.

## 3. Results

### 3.1. Samples and Patients

During the 25 years of the study, a total of 4757 samples from 2855 children were analyzed: 2593 exhaled air samples, 1065 gastroduodenal biopsies, 971 stool samples, and 128 string tests.

Of the 2855 children included in the study, 26.9% (*n* = 768) were infected with *H. pylori*, with 54.4% being female.

**Figure 1:**
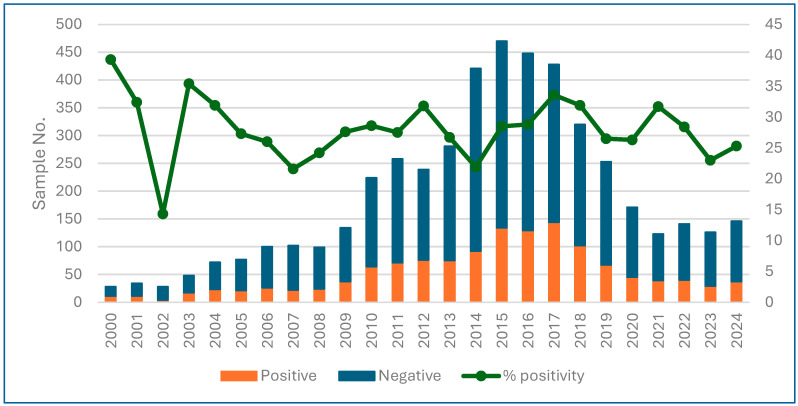
Samples analyzed and percentage of positivity per year (2000–2024) from patients < 15 years old, Gipuzkoa.

Between 2000 and 2009, 15% (*n* = 722) of the samples were received, with an average of 72 samples/year, but with an upward trend; between 2010 and 2019, 70% (*n* = 3342) of the samples were analyzed, exceeding 200 samples every year (range 224 to 470), and in the period 2020–2024, an average of 141 samples/year were received, constituting 15% of the samples. Global positivity by year is shown in Figure 1 and positivity by sample type (biopsy, UBT, stool, and string test) and by year can be found in the Supplementary Materials, Figures S1–S4, and the number of each type of sample received by year is in Figure S5.

Although there are variations in the number of samples received between the first and last years compared to the intermediate years, when the number of samples received was higher, there was no trend in positivity over the 25 years, which ranged from 22% to 35% (the first three years, when the number of samples was very small, were not taken into account).

Cases by age group are detailed in Table 2. The number of episodes increased with the age of the patients, with the highest number of episodes in the 12–14 age group (*n* = 325) and the lowest in the 0–2 age group (*n* = 7).

For the first diagnosis of the 2855 episodes, 3132 samples were received, with two or three different samples taken simultaneously from 266 children. The positivity of the tests for the initial diagnosis, considering only the first sample, was 30.4% for UBT (519/1703), 28.9% for the string test (26/90), 31.6% (184/582) for biopsy culture, and 17.6% (133/757) for stool antigen. There was a significant difference between the stool antigen tests compared to the other three tests (*p* < 0.001).

Of the total number of cases, 62.5% (480/768) underwent eradication control testing. No differences were observed between children who were diagnosed with invasive tests and those who were diagnosed with non-invasive tests (65.4% vs. 59.6%, *p* = 0.157). There was only one child with reinfection. In 2012, when he was 8 years old, he had a positive gastric biopsy culture for *H. pylori*. In 2016, without any diagnostic tests in between, he had another positive gastric biopsy culture. In 2017, he had a negative UBT, in 2018 a negative biopsy culture, and in 2019 a positive biopsy culture. The strains isolated in 2012 and 2016 were sensitive to all antimicrobials, while the strain isolated in 2019 was resistant to CLA and MTZ.

Table 3 shows the data of eradication control for age groups. The 12–14 age group may be biased because the study did not include tests on patients aged 15 and over, so controls may have been excluded in 14-year-old patients.

### 3.2. Antimicrobial Susceptibility Testing

**Figure 2:**
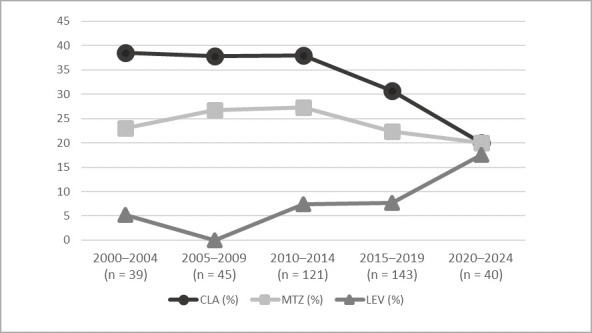
Evolution of overall resistance of *H. pylori* by five-year period in <15 years, Gipuzkoa, 2000–2024.

*H. pylori* was isolated in 400 samples (358 biopsies and 42 string tests), with 212 isolates obtained in the first sample received and 188 obtained from subsequent samples after eradication failures. Out of the 400 cultures positive *for H. pylori*, the antibiogram was obtained in 386 (96.5%) isolates. For CLA, data of susceptibility was obtained in 390 isolates; in 4 isolates, cultivation was not viable and susceptibility to CLA was obtained using molecular biology by sequencing the 23S gene. Susceptibility to RIF was studied in 41 isolates. All isolates were sensitive to AMX and TET. There was only one isolate resistant to RIF among the 41 studied. Overall, primary, and secondary resistance to CLA, MTZ, and LEV is shown in Table 4, and resistance by period is shown in Figure 2. Combined resistance to two antimicrobials was detected in 12.2% (47/386) of isolates and only three strains, 0.8%, were resistant to CLA, MTZ, and LEV.

## 4. Discussion

This study provides an accurate picture of the evolution over 25 years of the situation regarding antibiotic resistance and the use of diagnostic methods for *H. pylori* infection in children in a developed region with a high socioeconomic level. The fact that we have been able to analyze data for 25 years adds value to the study, as it allows us to monitor changes in the resistance profile and demonstrate that this is an issue in constant evolution, reinforcing the recommendation to maintain vigilance. In the early years of the study, the number of samples received was lower, probably because the techniques were being implemented and consolidated in the laboratory and in the clinical practice of pediatricians. In recent years, the number of samples has decreased, probably due to two causes: the COVID-19 pandemic and the update to the ESPHGAN and NASPGHAN guidelines. However, the percentage of positive results remained nearly stable.

Although invasive methods are recommended by pediatric guidelines for diagnosing *H. pylori* infection in children [4], in our study, non-invasive methods, UBT (59.6%) and stool antigen testing (26.5%), were used more frequently than endoscopy (23.5%). The influence and pressure from parents to have their children tested to rule out *H. pylori* infection must also be considered, and sometimes it may be challenging for the healthcare professional [15]. Manfredi et al. recommend initially using non-invasive tests in children with compatible clinical symptoms and using invasive tests to confirm infection [6].

UBT was the most commonly used test for the initial diagnosis, although the percentage of positive results (30.4%) was similar to that obtained with biopsy culture (31.6%) and the string test (28.9%), with the stool antigen test performing significantly worse (17.6%). Therefore, although stool samples are easy to collect in pediatrics, they have the disadvantage of giving false negatives for the diagnosis of *H. pylori*, e.g., in cases of constipation, low bacterial load, or uneven distribution of the antigen in the stool [16].

Gastric biopsies have been performed every year to rule out *H. pylori* infection. It is no longer acceptable to say that *H. pylori* culture is troublesome and time-consuming. It has many advantages over non-invasive tests, the main and most important being that it allows an antibiogram to be performed to provide targeted treatment. In our study, we were able to determine the susceptibility of 96.5% of the *H. pylori* isolates, which confirms that *H. pylori* culture is not that complicated. Furthermore, knowing the susceptibility pattern of *H. pylori* in pediatrics in the geographical area allows, in cases diagnosed by non-invasive methods, empirical treatment “adapted” to the known susceptibility, which increases the probability of *H. pylori* eradication.

To date, no AMX-resistant isolates have been detected in <15-year-olds in our region, and in adults, the figure has been minimal, 0.6% [12]. Botija et al. report 2.6% of resistant isolates in children in Madrid [7]. In contrast, in regions with different social and health characteristics, such as in South America and Asia, *H. pylori* resistance to AMX is 5% [17], and specific regions of Asia have even exceeded 70% [18].

Resistance to TET has not been detected in children in Gipuzkoa, and, exceptionally, is only 0.9% in Madrid [7]. However, global studies show a resistance in the Eastern Mediterranean of 3–5% [8,17]. Although we currently have no problems with resistance to these antimicrobials in our environment, migration and globalization mean that we must not neglect monitoring susceptibility to them.

In contrast, resistance to CLA was detected from the beginning of the study, and although the number of positive biopsies has decreased in the last period, resistance to CLA exceeded 15% in all periods of the study, which is the limit set by the guidelines for empirical use of this antimicrobial. Therefore, whenever possible, an invasive test should be performed to determine susceptibility and provide targeted treatment.

Resistance to CLA is not uniform across Europe, but except in Belgium, where Kotilea reports primary resistance of 11% [19], in the rest of Europe, with health and social characteristics similar to the region where this study was conducted, resistance to CLA in children is higher than 15% [8]. Specifically, in Madrid, it was 38.2% [7]. In adults in our region, primary resistance to CLA was around 14% [12] for the same period, lower than in children. This is probably because macrolides are used very frequently in pediatrics to treat other infections, especially respiratory infections.

Resistance to LEV has increased considerably in the period 2019–2024 compared to the previous period, from 7.5% to 17.5%. Megraud et al. in a European study explain this resistance through the increased use of quinolones for other infectious diseases [20]. Quinolones are restricted for use in children, but LEV-resistant isolates may have been transmitted to children through their parents who may require this antibiotic.

As for MTZ, the rate of primary resistance in our study has remained constant over the years, 19.4%, similar to the 17.8% reported by Botija et al. in Madrid [7], and also similar to that reported for Europe by Salahi-Niri et al., but considerably lower than that observed in the other WHO Regions: the Americas, Eastern Mediterranean, and Eastern Pacific, where resistance exceeds 40% [8]. Altay et al. report an MTZ resistance level for Turkey of 77.5% [21]. In the case of MTZ, in vitro resistance does not always translate to clinical failure, and reproducibility of the technique is low. Due to this, and the lack of alternative techniques for the detection of resistance like molecular biology methods, No. 19 of the ESPHGAN guidelines recommends not using MTZ susceptibility data to choose eradication therapy [4].

Secondary resistance to both CLA and MTZ was higher than primary resistance, at around 40% and 30%, respectively. These are first-line antibiotics, which would support the theory of selection of resistant mutants after initial treatment. Therefore, susceptibility testing is required before treating children who have previously failed eradication therapy.

In our study, eradication monitoring was performed in 62.5% (480/768) of cases using UBT or stool antigen. The guidelines recommend always performing an eradication control test, and the UBT and stool antigen detection are the recommended tests for this purpose [4]. One possible explanation for not performing it could be that patients had clinical resolution and did not return to the pediatrician. Among the 480 children with post-treatment follow-up, *H. pylori* eradication was achieved in 75.7% (103/136) of those diagnosed by invasive methods and in 48.0% (165/344) of those diagnosed by non-invasive methods. This difference was statistically significant (*p* < 0.0001) and supports the use of invasive diagnostic approaches that allow antimicrobial susceptibility testing and, consequently, targeted therapy.

In our study, reinfection was anecdotal; in 25 years, we detected only one case of reinfection. The explanation may lie in the fact that the study was conducted in a region with a high socioeconomic and health level, conditions contrary to those described by Hu Y et al. as favoring *H. pylori* recurrences and reinfections [22].

The study has limitations. First, the number of *H. pylori* isolates studied in the first and last five-year periods is low. This would be positive if it reflected a decrease in *H. pylori* infections in pediatrics, but we fear that it is rather a reflection of a lack of diagnosis. This is probably because parents and pediatricians are reticent to perform an endoscopy with sedation on a child, due to its inherent risks. Second, as this is a retrospective, long-term study, there may be patients for whom follow-up could not be completed due to a change of residence or because they reached the age of 15 during the study and their data could not be collected. Finally, the data is from just one region of Spain. This does not necessarily make it any less useful, as it is still valuable for treating the local pediatric population. Our results may also reflect what is happening in the rest of the country, given that there are no major differences in socioeconomic and hygiene conditions from other autonomous communities.

In conclusion, the high levels of resistance, both primary and secondary, to clarithromycin (28.9% and 38.7%) and metronidazole (19.4% and 29.7%) make it essential that treatment of *H. pylori* infection in pediatrics be targeted. The initial diagnosis could be made with non-invasive tests, but if these are positive, an endoscopy would be necessary to isolate the strain and perform antimicrobial susceptibility studies. This would greatly facilitate the clinical management of *H. pylori* infection in children for pediatricians and gastroenterologists.

## Figures and Tables

**Table 1:** Population under 15 years of age attended to at Donostia University Hospital.

Year	2001	2006	2011	2016	2020	2024
Population < 15 years	49,123	53,319	59,172	59,990	58,314	54,196

**Table 2:** *H. pylori* episodes and positive diagnostic tests of the primary samples by age group in <15 years, Gipuzkoa, 2000–2024.

Age (Years)	No. of Cases	Culture-Positive No. (%)	UBT-Positive No. (%)	Antigen-Positive No. (%)
0–2	7	2 (28.6)	4 (57.1)	1 (14.3)
3–5	49	13 (26.5)	23 (46.9)	17 (34.7)
6–8	136	28 (20.6)	96 (70.6)	30 (22.1)
9–11	251	71 (28.3)	162 (64.5)	56 (22.3)
12–14	325	98 (30.2)	236 (72.6)	47 (14.5)
Total	768	212 (27.6)	521 (67.8)	151 (19.7)

**Table 3:** Control of *H. pylori* episodes by age group in children < 15 years, Gipuzkoa, 2000–2024.

Age (Years)	No. Cases	Post-Treatment Control			No Post-Treatment Control
		Total No. (%)	Eradicated Cases No. (%)	Non-Eradicated Cases No. (%)	Cases No. (%)
0–2	7	3 (42.9)	1 (33.3)	2 (66.7)	4 (57.1)
3–5	49	27 (55.1)	12 (44.4)	15 (55.6)	22 (44.9)
6–8	136	93 (68.4)	48 (51.6)	45 (48.4)	43 (31.6)
9–11	251	164 (65.3)	90 (54.9)	74 (45.1)	87 (34.7)
12–14	325	193 (59.4)	117 (60.6)	76 (39.4)	132 (40.6) *
Total	768	480 (62.5)	268 (55.8)	212 (44.2)	288 (37.5)

* In total, 74 patients were 14 years old, and there is no data on whether those aged ≥ 15 were monitored.

**Table 4:** Resistance of 386 *H. pylori* isolates from children < 15 years, Gipuzkoa, 2000–2024.

	Overall Resistance (*n* = 386)	Primary Resistance (*n* = 201)	Secondary Resistance (*n* = 185)	*p* Value **
CLA *	33.6%	28.9%	38.7%	0.042
MTZ	24.4%	19.4%	29.7%	0.024
LEV	7.5%	8.9%	5.9%	0.33

CLA = clarithromycin, MTZ = metronidazole, LEV = levofloxacin. * For CLA *n* = 390, 4 isolates included in primary resistance were analyzed by molecular biology. ** *p* compares primary resistance to secondary resistance in each antimicrobial agent.

## Data Availability

The data presented in this study are available on request from the corresponding author. The data are not publicly available due to privacy reasons.

## Supplementary Materials

The following supporting information can be downloaded at https://www.mdpi.com/article/10.3390/microorganisms14020389/s1, Figure S1: Number of urea breath tests (UBTs) per year (2000–2024) from patients < 15 years old, Gipuzkoa; Figure S2: Number of gastroduodenal biopsies per year (2000–2024) from patients < 15 years old, Gipuzkoa; Figure S3: Number of string tests (Entero-tests) per year (2000–2013) from patients < 15 years old, Gipuzkoa; Figure S4: Number of stool antigen tests per year (2014–2024) from patients < 15 years old, Gipuzkoa; Figure S5: Number of each type of sample received by year from patients < 15 years old, Gipuzkoa.
