# Supplementary material for: Helicobacter pylori Infection and Antimicrobial Resistance Surveillance over 25 Years in Children in Gipuzkoa, Northern Spain
Source: Microorganisms. 2026 Feb 6;14(2):389. doi: 10.3390/microorganisms14020389 (PMC12943096; doi:10.3390/microorganisms14020389)

Supplementary Figure S1. Number of urea breath test (UBT) per year (2000-2024) from patients <15 years old, Gipuzkoa

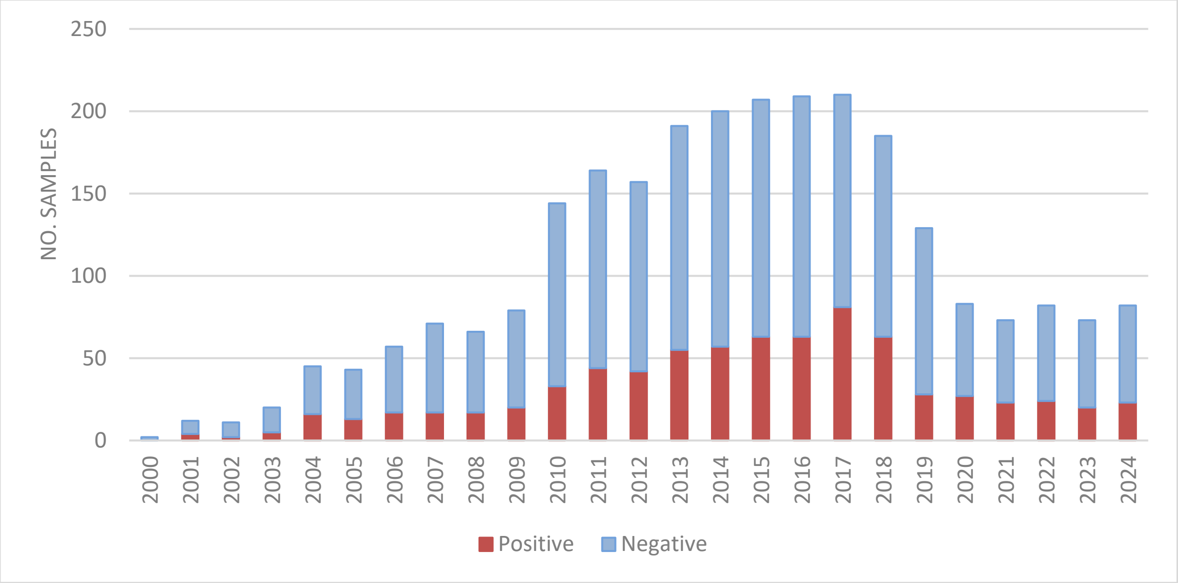

Supplementary Figure S2. Number of gastroduodenal biopsies per year (2000-2024) from patients <15 years old, Gipuzkoa

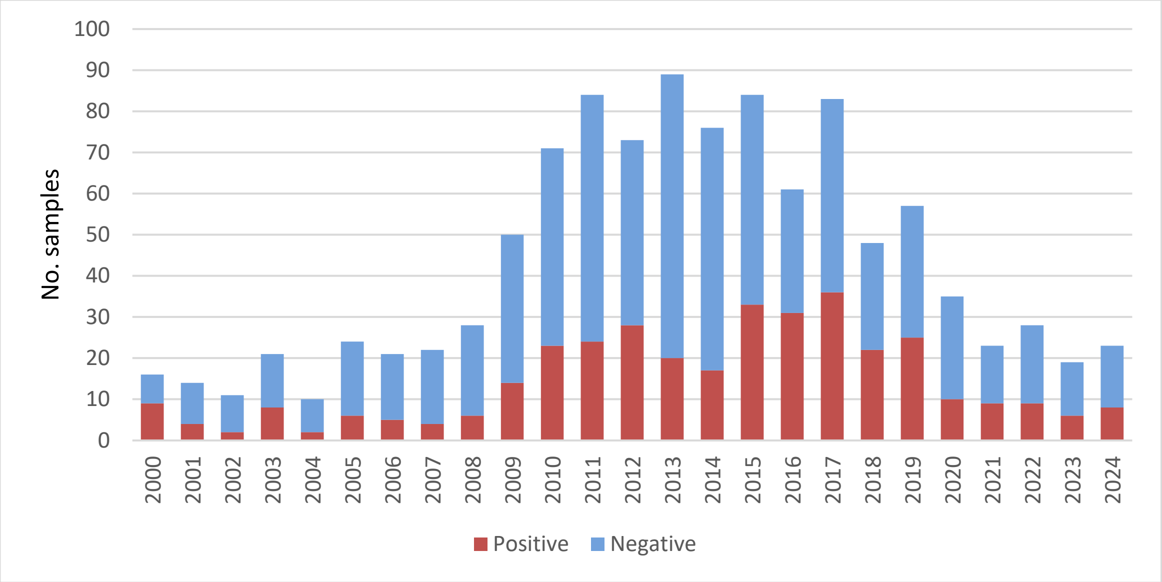

Supplementary Figure S3. Number of string test (Entero-test) per year (2000-2013) from patients <15 years old, Gipuzkoa

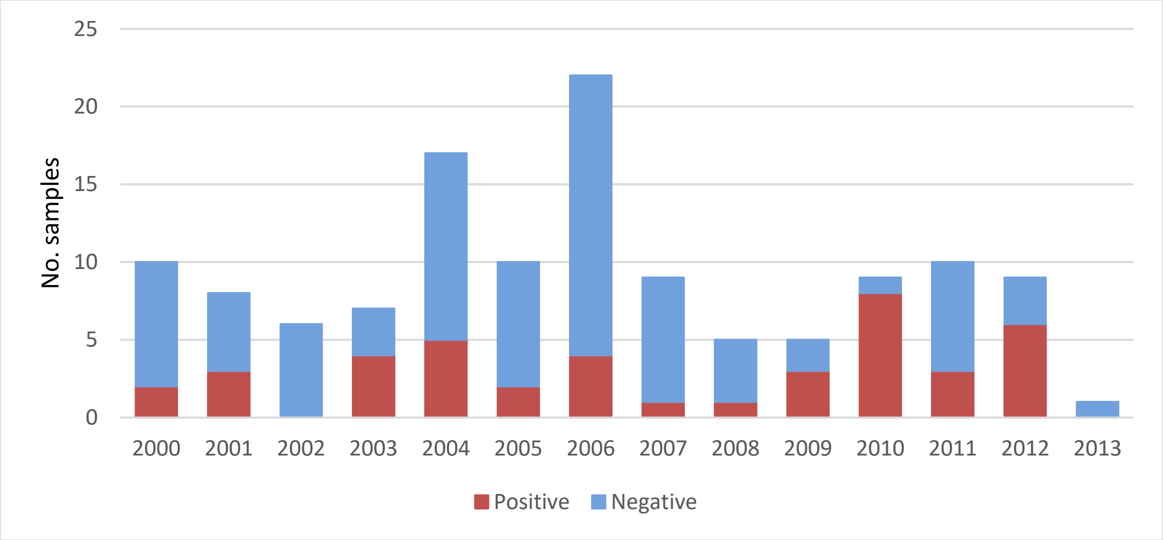

Supplementary Figure S4. Number of stool antigen test per year (2014-2024) from patients <15 years old, Gipuzkoa

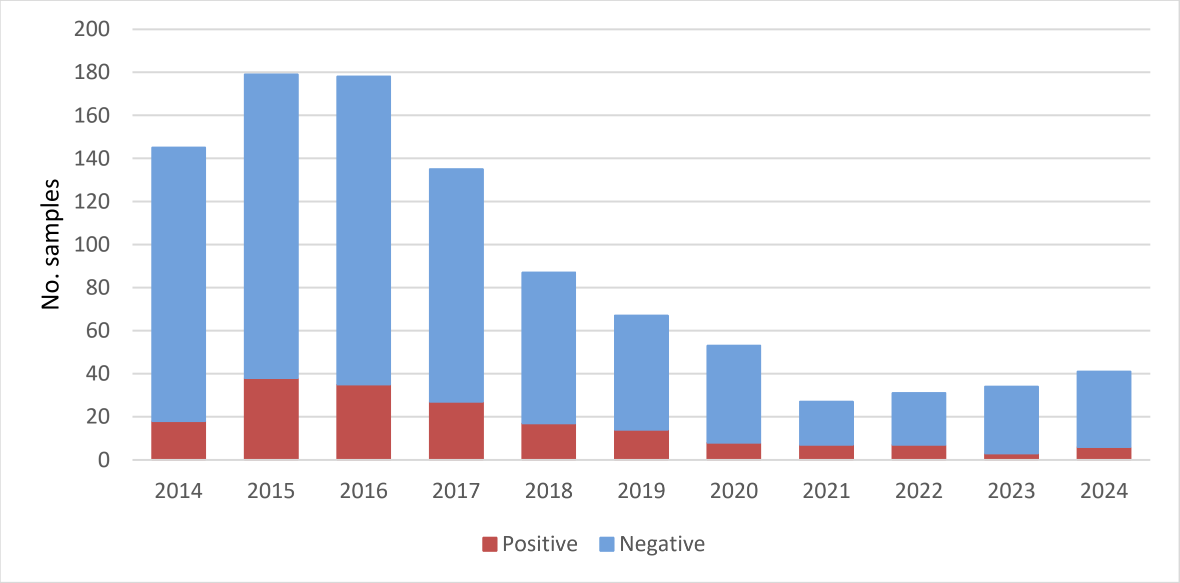

Supplementary Figure S5. Number of each type of sample received by year from patients <15 years old, Gipuzkoa.

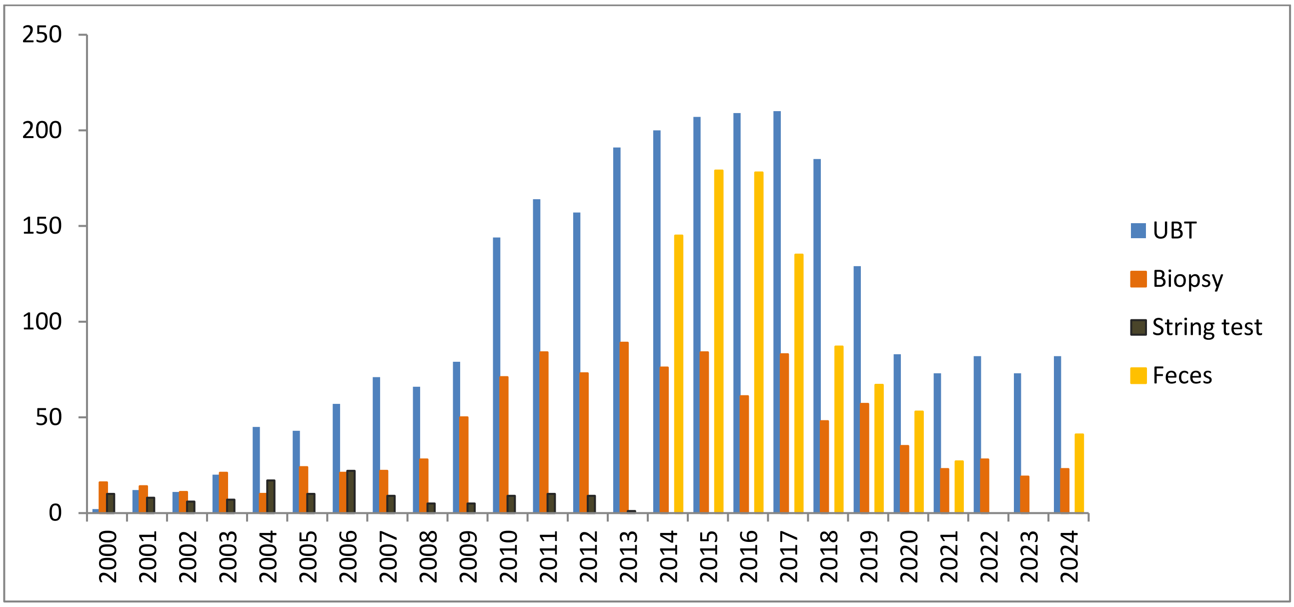

Supplement: Supplementary file 1 [file microorganisms-14-00389-s001.zip › microorganisms-4091382-supplementary.pdf]
